# 1-Methyl-3-(2-methyl­phen­yl)-3a-nitro-1,2,3,3a,4,9b-hexa­hydro­chromeno[4,3-*b*]pyrrole

**DOI:** 10.1107/S1600536812020338

**Published:** 2012-05-16

**Authors:** S. Sundaramoorthy, N. Sivakumar, M. Bakthadoss, D. Velmurugan

**Affiliations:** a Centre of Advanced Study in Crystallography and Biophysics, University of Madras, Guindy Campus, Chennai 600 025, India; b Department of Organic Chemistry, University of Madras, Guindy Campus, Chennai 600 025, India

## Abstract

The asymmetric unit of the title compound, C_19_H_20_N_2_O_3_, contains two independent mol­ecules in both of which the pyrrolidine ring adopts an envelope conformation, but with a C atom as the flap in one mol­ecule and the N atom in the other. The pyran ring adopts a half-chair conformation in both mol­ecules. In the crystal, mol­ecules are linked *via* C—H⋯O hydrogen bonds and C—H⋯π inter­actions.

## Related literature

For a related structure, see: Chitra Devi *et al.* (2011 ▶). For ring conformations, see: Cremer & Pople (1975 ▶).
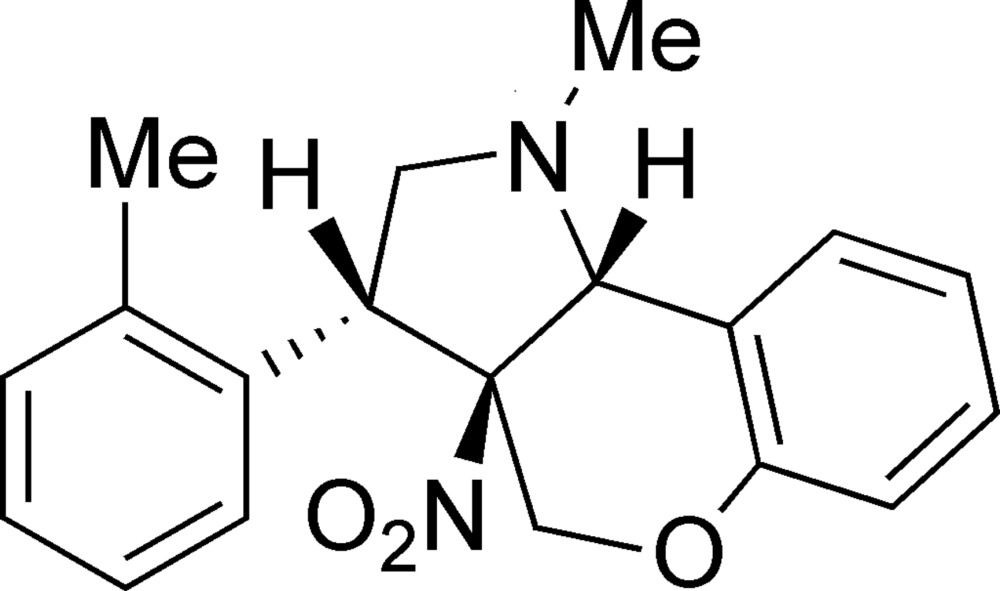


## Experimental

### 

#### Crystal data

C_19_H_20_N_2_O_3_
*M* *_r_* = 324.37
Monoclinic, 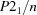

*a* = 17.8782 (13) Å
*b* = 8.0447 (6) Å
*c* = 23.4660 (17) Å
β = 97.605 (2)°
*V* = 3345.3 (4) Å^3^
*Z* = 8
Mo *K*α radiation
μ = 0.09 mm^−1^
*T* = 293 K
0.24 × 0.23 × 0.2 mm

#### Data collection

Bruker SMART APEXII area-detector diffractometer
Absorption correction: multi-scan (*SADABS*; Bruker, 2008 ▶) *T*_min_ = 0.979, *T*_max_ = 0.983
30608 measured reflections
8190 independent reflections
5303 reflections with *I* > 2σ(*I*)
*R*_int_ = 0.030

#### Refinement

*R*[*F* ^2^ > 2σ(*F* ^2^)] = 0.049
*wR*(*F* ^2^) = 0.149
*S* = 1.00
8190 reflections
437 parameters
H-atom parameters constrained
Δρ_max_ = 0.20 e Å^−3^
Δρ_min_ = −0.24 e Å^−3^

### 

Data collection: *APEX2* (Bruker, 2008 ▶); cell refinement: *SAINT* (Bruker, 2008 ▶); data reduction: *SAINT*; program(s) used to solve structure: *SHELXS97* (Sheldrick, 2008 ▶); program(s) used to refine structure: *SHELXL97* (Sheldrick, 2008 ▶); molecular graphics: *ORTEP-3* (Farrugia, 1997 ▶); software used to prepare material for publication: *SHELXL97* and *PLATON* (Spek, 2009 ▶).

## Supplementary Material

Crystal structure: contains datablock(s) global, I. DOI: 10.1107/S1600536812020338/bt5906sup1.cif

Structure factors: contains datablock(s) I. DOI: 10.1107/S1600536812020338/bt5906Isup2.hkl

Supplementary material file. DOI: 10.1107/S1600536812020338/bt5906Isup3.cml

Additional supplementary materials:  crystallographic information; 3D view; checkCIF report

## Figures and Tables

**Table 1:** Hydrogen-bond geometry (Å, °) *Cg*11 and *Cg*3 are the centroids of the C12′–C17′ and C1–C6 rings, respectively.

*D*—H⋯*A*	*D*—H	H⋯*A*	*D*⋯*A*	*D*—H⋯*A*
C15—H15⋯O1′^i^	0.93	2.60	3.282 (3)	131
C4′—H4′⋯O2^ii^	0.93	2.56	3.407 (3)	152
C14′—H14′⋯O2	0.93	2.59	3.327 (2)	136
C19′—H19*F*⋯O1′	0.96	2.54	3.391 (3)	148
C18—H18*A*⋯*Cg*11^i^	0.96	2.92	3.854 (2)	166
C19′—H19*E*⋯*Cg*3^iii^	0.96	2.94	3.811 (3)	152

Symmetry codes: (i) 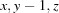; (ii) 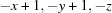; (iii) 
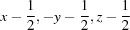
.

**Fig. 1.**
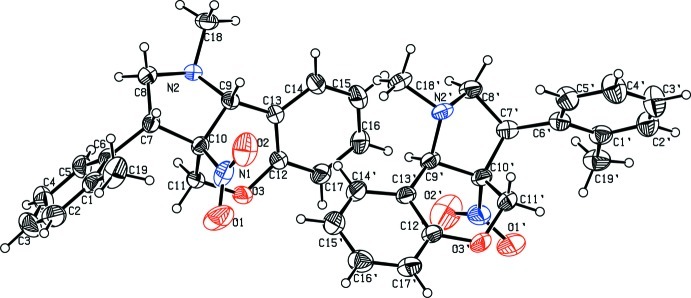
A perspective view of the molecule showing displacement ellipsoids drawn at the 30% probability level.

**Fig. 2.**
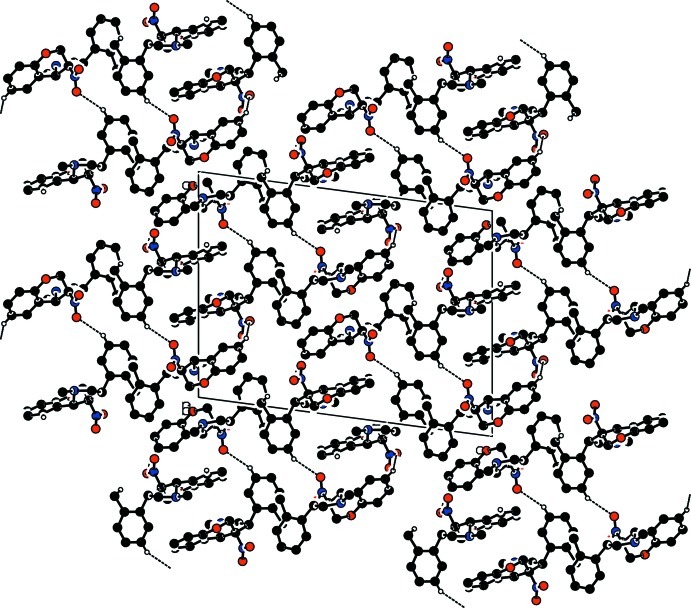
C—H···O interactions (dotted lines) in the crystal structure of the title compound. The crystal packing of the molecules is viewed down the b axis.

## supplementary crystallographic information

### Comment

In order to obtain detailed information on its molecular conformation, the X-ray structure of the title compound has been determined and is discussed here.

The geometric parameters of the title molecule (Fig. 1) agree well with reported similar structure (Chitra Devi *et al.*, 2011). The pyrrolidine rings make the dihedral angle of 54.29 (9)° and 40.05 (9)° with benzene rings of chromeno moiety for both the molecules. In both the molecules, the average dihedral angle between methyl benzene ring and pyrrolidine ring is 66.83 (9)°. The sum of bond angles around N1 [331.9 (3)°, 329.15 (4)°] and around N2 atom [359.9 (5)°, 360.2 (5)°] indicate *sp*^3^ and *sp*^2^ hybridizations in two molecules, respectively.

The pyran ring adopts half-chair conformation, with the puckering parameters (Cremer & Pople, 1975) of q_2_ = 0.3472 (16) Å, q_3_ = 0.2945 (15) Å, φ = 97.6 (2)°, in one molecule and in the other molecule q_2_ = 0.3655 (17) Å, q_3_ = 0.2930 (17) Å, φ = 77.4 (2)°. The crystal packing is stabilized by C—H···O and C—H···π interactions. Atom C15 donating one proton to O1' at (*x*, *y* - 1, *z*) and the C4' atom in the other molecule donating one proton to O2 at (-*x* + 1, -*y* + 1, -*z*) form C—H···O hydrogen bonds.

### Experimental

A mixture of (2E)-methyl-2-(2-formylphenoxy)methyl-3-phenylacrylate (2 mmol, 0.592 g) and sarcosine (2 mmol, 0.178 g) in acetonitrile (8 ml) was refluxed for 5 h. After the completion of the reaction as indicated by TLC, the reaction mixture was concentrated and the resulting crude mass was diluted with water (15 ml) and extracted with ethyl acetate (3 *X* 15 ml). The combined organic layer obtained was washed with brine (2 *X* 10 ml) and dried over anhydrous Na2SO4. The organic layer was concentrated and purified by column chromatography on silica gel (Acme 100–200 mesh), ethyl acetate: hexane (1: 9) to afford the pure methyl 1,2,3,3a,4,9 b-hexahydro-1-methyl-3-phenyl chromeno[4,3-*b*] pyrro-le-3a-carboxytate as colourless solid in 91% (0.588 g) yieid.

### Refinement

H atoms were positioned geometrically (C—H = 0.93–0.98 Å) and allowed to ride on their parent atoms, with 1.5*U*_eq_(C) for methyl H and 1.2 *U*_eq_(C) for other H atoms.

### Crystal data

**Table:**

C_19_H_20_N_2_O_3_	*F*(000) = 1376
*M**_r_* = 324.37	*D*_x_ = 1.288 Mg m^−^^3^
Monoclinic, *P*2_1_/*n*	Mo *K*α radiation, λ = 0.71073 Å
Hall symbol: -P 2yn	Cell parameters from 1223 reflections
*a* = 17.8782 (13) Å	θ = 1.4–28.4°
*b* = 8.0447 (6) Å	µ = 0.09 mm^−^^1^
*c* = 23.4660 (17) Å	*T* = 293 K
β = 97.605 (2)°	Block, colourless
*V* = 3345.3 (4) Å^3^	0.24 × 0.23 × 0.2 mm
*Z* = 8	

### Data collection

**Table:**

Bruker SMART APEXII area-detector diffractometer	8190 independent reflections
Radiation source: fine-focus sealed tube	5303 reflections with *I* > 2σ(*I*)
Graphite monochromator	*R*_int_ = 0.030
ω and φ scans	θ_max_ = 28.4°, θ_min_ = 1.4°
Absorption correction: multi-scan (*SADABS*; Bruker, 2008)	*h* = −23→23
*T*_min_ = 0.979, *T*_max_ = 0.983	*k* = −10→10
30608 measured reflections	*l* = −31→31

### Refinement

**Table:**

Refinement on *F*^2^	Primary atom site location: structure-invariant direct methods
Least-squares matrix: full	Secondary atom site location: difference Fourier map
*R*[*F*^2^ > 2σ(*F*^2^)] = 0.049	Hydrogen site location: inferred from neighbouring sites
*wR*(*F*^2^) = 0.149	H-atom parameters constrained
*S* = 1.00	*w* = 1/[σ^2^(*F*_o_^2^) + (0.0745*P*)^2^ + 0.4617*P*] where *P* = (*F*_o_^2^ + 2*F*_c_^2^)/3
8190 reflections	(Δ/σ)_max_ < 0.001
437 parameters	Δρ_max_ = 0.20 e Å^−^^3^
0 restraints	Δρ_min_ = −0.24 e Å^−^^3^

### Special details

**Table:**

Geometry. All e.s.d.'s (except the e.s.d. in the dihedral angle between two l.s. planes) are estimated using the full covariance matrix. The cell e.s.d.'s are taken into account individually in the estimation of e.s.d.'s in distances, angles and torsion angles; correlations between e.s.d.'s in cell parameters are only used when they are defined by crystal symmetry. An approximate (isotropic) treatment of cell e.s.d.'s is used for estimating e.s.d.'s involving l.s. planes.
Refinement. Refinement of *F*^2^ against ALL reflections. The weighted *R*-factor *wR* and goodness of fit *S* are based on *F*^2^, conventional *R*-factors *R* are based on *F*, with *F* set to zero for negative *F*^2^. The threshold expression of *F*^2^ > σ(*F*^2^) is used only for calculating *R*-factors(gt) *etc*. and is not relevant to the choice of reflections for refinement. *R*-factors based on *F*^2^ are statistically about twice as large as those based on *F*, and *R*- factors based on ALL data will be even larger.

### Fractional atomic coordinates and isotropic or equivalent isotropic displacement parameters (Å^2^)

**Table:**

	*x*	*y*	*z*	*U*_iso_*/*U*_eq_	
C1	0.02218 (11)	0.2127 (2)	−0.20011 (6)	0.0595 (4)	
C2	−0.04495 (16)	0.2582 (3)	−0.23344 (8)	0.0828 (7)	
H2	−0.0427	0.3143	−0.2678	0.099*	
C3	−0.11375 (15)	0.2233 (3)	−0.21748 (9)	0.0950 (8)	
H3	−0.1575	0.2571	−0.2405	0.114*	
C4	−0.11849 (11)	0.1391 (3)	−0.16774 (8)	0.0814 (6)	
H4	−0.1653	0.1120	−0.1572	0.098*	
C5	−0.05315 (9)	0.0942 (2)	−0.13315 (7)	0.0579 (4)	
H5	−0.0566	0.0378	−0.0990	0.070*	
C6	0.01721 (8)	0.13101 (18)	−0.14794 (6)	0.0453 (3)	
C7	0.08806 (8)	0.08815 (19)	−0.10752 (6)	0.0451 (3)	
H7	0.1301	0.0877	−0.1304	0.054*	
C8	0.08968 (10)	−0.0800 (2)	−0.07584 (6)	0.0566 (4)	
H8A	0.0398	−0.1295	−0.0804	0.068*	
H8B	0.1241	−0.1562	−0.0911	0.068*	
C9	0.15975 (7)	0.10856 (19)	−0.01373 (6)	0.0421 (3)	
H9	0.2080	0.0871	−0.0279	0.051*	
C10	0.10890 (8)	0.21210 (18)	−0.05732 (6)	0.0430 (3)	
C11	0.04311 (8)	0.2785 (2)	−0.03001 (6)	0.0478 (4)	
H11A	0.0113	0.3449	−0.0580	0.057*	
H11B	0.0132	0.1859	−0.0192	0.057*	
C12	0.12604 (8)	0.32064 (19)	0.05721 (6)	0.0456 (3)	
C13	0.17300 (8)	0.19351 (19)	0.04372 (6)	0.0437 (3)	
C14	0.23511 (9)	0.1557 (2)	0.08367 (7)	0.0633 (5)	
H14	0.2687	0.0740	0.0751	0.076*	
C15	0.24807 (12)	0.2370 (3)	0.13589 (8)	0.0778 (6)	
H15	0.2894	0.2083	0.1624	0.093*	
C16	0.19997 (11)	0.3598 (3)	0.14866 (7)	0.0712 (5)	
H16	0.2085	0.4139	0.1839	0.085*	
C17	0.13908 (10)	0.4035 (2)	0.10942 (7)	0.0586 (4)	
H17	0.1068	0.4880	0.1178	0.070*	
C18	0.15600 (10)	−0.1859 (2)	0.01335 (7)	0.0574 (4)	
H18A	0.2014	−0.2047	−0.0035	0.086*	
H18B	0.1248	−0.2834	0.0086	0.086*	
H18C	0.1686	−0.1622	0.0536	0.086*	
C19	0.09576 (14)	0.2474 (3)	−0.22223 (8)	0.0878 (7)	
H19A	0.1217	0.1447	−0.2265	0.132*	
H19B	0.1264	0.3176	−0.1955	0.132*	
H19C	0.0862	0.3022	−0.2588	0.132*	
N1	0.11522 (6)	−0.04540 (15)	−0.01501 (5)	0.0435 (3)	
N2	0.15168 (10)	0.3563 (2)	−0.07917 (5)	0.0635 (4)	
O1	0.11776 (11)	0.48303 (19)	−0.09268 (7)	0.1014 (5)	
O2	0.21817 (9)	0.3339 (2)	−0.08460 (7)	0.0966 (5)	
O3	0.06649 (6)	0.37738 (15)	0.01962 (4)	0.0589 (3)	
C1'	0.55207 (10)	0.7413 (2)	0.25226 (7)	0.0575 (4)	
C2'	0.62524 (12)	0.7853 (3)	0.27581 (8)	0.0763 (6)	
H2'	0.6326	0.8360	0.3117	0.092*	
C3'	0.68625 (12)	0.7563 (4)	0.24788 (10)	0.0930 (7)	
H3'	0.7343	0.7873	0.2646	0.112*	
C4'	0.67598 (11)	0.6809 (3)	0.19500 (9)	0.0865 (7)	
H4'	0.7172	0.6615	0.1755	0.104*	
C5'	0.60464 (10)	0.6338 (3)	0.17073 (7)	0.0665 (5)	
H5'	0.5983	0.5821	0.1350	0.080*	
C6'	0.54199 (9)	0.6622 (2)	0.19867 (6)	0.0516 (4)	
C7'	0.46479 (9)	0.6026 (2)	0.17122 (7)	0.0526 (4)	
H7'	0.4334	0.5847	0.2019	0.063*	
C8'	0.46583 (10)	0.4408 (2)	0.13662 (7)	0.0598 (4)	
H8'1	0.5135	0.3833	0.1461	0.072*	
H8'2	0.4253	0.3675	0.1443	0.072*	
C9'	0.39224 (8)	0.61203 (18)	0.07478 (6)	0.0468 (3)	
H9'	0.3470	0.5529	0.0832	0.056*	
C10'	0.42164 (8)	0.72296 (19)	0.12586 (7)	0.0503 (4)	
C11'	0.47021 (10)	0.8579 (2)	0.10545 (8)	0.0618 (4)	
H11C	0.4893	0.9281	0.1377	0.074*	
H11D	0.5131	0.8076	0.0907	0.074*	
C12'	0.39385 (9)	0.8719 (2)	0.01524 (7)	0.0526 (4)	
C13'	0.37439 (8)	0.70552 (19)	0.01904 (7)	0.0478 (3)	
C14'	0.33460 (9)	0.6325 (2)	−0.02933 (7)	0.0607 (4)	
H14'	0.3195	0.5222	−0.0275	0.073*	
C15'	0.31695 (11)	0.7189 (3)	−0.07995 (8)	0.0711 (5)	
H15'	0.2916	0.6663	−0.1122	0.085*	
C16'	0.33701 (11)	0.8836 (3)	−0.08254 (8)	0.0721 (5)	
H16'	0.3250	0.9429	−0.1165	0.086*	
C17'	0.37472 (10)	0.9603 (2)	−0.03502 (8)	0.0648 (5)	
H17'	0.3874	1.0722	−0.0366	0.078*	
C18'	0.44213 (11)	0.3545 (2)	0.03745 (8)	0.0638 (5)	
H18D	0.4828	0.2763	0.0449	0.096*	
H18E	0.4392	0.3937	−0.0014	0.096*	
H18F	0.3955	0.3013	0.0429	0.096*	
C19'	0.48834 (12)	0.7791 (3)	0.28559 (8)	0.0723 (5)	
H19D	0.5081	0.8218	0.3227	0.108*	
H19E	0.4603	0.6792	0.2901	0.108*	
H19F	0.4557	0.8603	0.2653	0.108*	
N1'	0.45555 (7)	0.49419 (15)	0.07680 (6)	0.0503 (3)	
N2'	0.35723 (10)	0.8038 (2)	0.15211 (7)	0.0708 (4)	
O1'	0.36829 (10)	0.9366 (2)	0.17476 (8)	0.1120 (6)	
O2'	0.29936 (10)	0.7331 (3)	0.15009 (11)	0.1409 (9)	
O3'	0.42965 (8)	0.95748 (14)	0.06165 (5)	0.0702 (4)	

### Atomic displacement parameters (Å^2^)

**Table:**

	*U*^11^	*U*^22^	*U*^33^	*U*^12^	*U*^13^	*U*^23^
C1	0.0953 (12)	0.0420 (9)	0.0390 (7)	−0.0038 (9)	0.0005 (8)	−0.0055 (7)
C2	0.137 (2)	0.0639 (13)	0.0399 (8)	0.0232 (14)	−0.0154 (11)	−0.0052 (8)
C3	0.1028 (17)	0.112 (2)	0.0596 (12)	0.0460 (15)	−0.0303 (12)	−0.0294 (12)
C4	0.0604 (10)	0.1124 (18)	0.0670 (11)	0.0170 (11)	−0.0083 (9)	−0.0338 (12)
C5	0.0572 (9)	0.0653 (11)	0.0493 (8)	0.0002 (8)	−0.0003 (7)	−0.0102 (8)
C6	0.0576 (8)	0.0379 (8)	0.0386 (7)	−0.0013 (7)	−0.0003 (6)	−0.0057 (6)
C7	0.0500 (8)	0.0423 (8)	0.0422 (7)	−0.0030 (7)	0.0029 (6)	−0.0060 (6)
C8	0.0681 (10)	0.0417 (9)	0.0557 (9)	0.0025 (8)	−0.0074 (7)	−0.0034 (7)
C9	0.0373 (7)	0.0445 (8)	0.0444 (7)	0.0040 (6)	0.0046 (5)	−0.0028 (6)
C10	0.0505 (8)	0.0381 (8)	0.0396 (7)	−0.0048 (6)	0.0027 (6)	0.0006 (6)
C11	0.0508 (8)	0.0433 (8)	0.0463 (7)	0.0108 (7)	−0.0050 (6)	−0.0044 (6)
C12	0.0487 (8)	0.0437 (8)	0.0427 (7)	0.0020 (7)	0.0001 (6)	−0.0002 (6)
C13	0.0426 (7)	0.0434 (8)	0.0435 (7)	0.0007 (6)	0.0002 (6)	−0.0010 (6)
C14	0.0545 (9)	0.0682 (12)	0.0620 (9)	0.0129 (9)	−0.0115 (7)	−0.0090 (9)
C15	0.0794 (12)	0.0815 (14)	0.0628 (11)	0.0130 (11)	−0.0267 (9)	−0.0120 (10)
C16	0.0909 (13)	0.0687 (12)	0.0485 (9)	0.0011 (11)	−0.0121 (9)	−0.0154 (9)
C17	0.0725 (10)	0.0512 (10)	0.0505 (8)	0.0064 (8)	0.0025 (7)	−0.0093 (7)
C18	0.0648 (10)	0.0470 (9)	0.0595 (9)	0.0186 (8)	0.0050 (7)	0.0040 (8)
C19	0.1280 (18)	0.0886 (16)	0.0490 (9)	−0.0409 (14)	0.0193 (11)	0.0009 (10)
N1	0.0453 (6)	0.0355 (6)	0.0481 (6)	0.0064 (5)	0.0005 (5)	0.0005 (5)
N2	0.0918 (11)	0.0550 (10)	0.0434 (7)	−0.0256 (9)	0.0074 (7)	−0.0029 (7)
O1	0.1657 (16)	0.0489 (8)	0.0896 (10)	−0.0124 (10)	0.0174 (10)	0.0192 (8)
O2	0.0892 (10)	0.1105 (13)	0.0938 (10)	−0.0478 (10)	0.0260 (8)	0.0015 (9)
O3	0.0655 (7)	0.0546 (7)	0.0520 (6)	0.0232 (6)	−0.0089 (5)	−0.0131 (5)
C1'	0.0753 (11)	0.0482 (9)	0.0495 (8)	0.0097 (8)	0.0102 (8)	0.0058 (7)
C2'	0.0899 (14)	0.0777 (14)	0.0581 (10)	−0.0002 (11)	−0.0018 (9)	−0.0064 (10)
C3'	0.0700 (13)	0.122 (2)	0.0840 (14)	−0.0153 (13)	−0.0001 (11)	−0.0111 (14)
C4'	0.0569 (11)	0.121 (2)	0.0831 (13)	−0.0001 (12)	0.0162 (9)	−0.0044 (13)
C5'	0.0613 (10)	0.0808 (13)	0.0586 (9)	0.0073 (10)	0.0120 (8)	−0.0063 (9)
C6'	0.0564 (9)	0.0486 (9)	0.0504 (8)	0.0075 (7)	0.0094 (7)	0.0045 (7)
C7'	0.0547 (8)	0.0450 (9)	0.0597 (9)	0.0052 (7)	0.0134 (7)	0.0044 (7)
C8'	0.0667 (10)	0.0389 (8)	0.0731 (10)	0.0055 (8)	0.0069 (8)	0.0052 (8)
C9'	0.0464 (8)	0.0317 (7)	0.0635 (9)	−0.0029 (6)	0.0116 (6)	−0.0035 (7)
C10'	0.0513 (8)	0.0364 (8)	0.0631 (9)	0.0057 (7)	0.0079 (7)	−0.0049 (7)
C11'	0.0714 (10)	0.0366 (8)	0.0728 (10)	−0.0094 (8)	−0.0074 (8)	0.0014 (8)
C12'	0.0550 (9)	0.0368 (8)	0.0657 (9)	0.0025 (7)	0.0064 (7)	−0.0025 (7)
C13'	0.0453 (7)	0.0366 (8)	0.0621 (9)	0.0034 (6)	0.0089 (6)	−0.0047 (7)
C14'	0.0602 (10)	0.0477 (10)	0.0731 (11)	−0.0001 (8)	0.0045 (8)	−0.0128 (9)
C15'	0.0782 (12)	0.0709 (13)	0.0621 (10)	0.0071 (10)	0.0010 (9)	−0.0137 (10)
C16'	0.0832 (13)	0.0721 (13)	0.0612 (10)	0.0139 (11)	0.0107 (9)	0.0044 (10)
C17'	0.0777 (11)	0.0460 (9)	0.0713 (11)	0.0043 (9)	0.0119 (9)	0.0064 (9)
C18'	0.0774 (11)	0.0334 (8)	0.0827 (12)	0.0009 (8)	0.0189 (9)	−0.0100 (8)
C19'	0.0963 (14)	0.0680 (12)	0.0558 (9)	0.0215 (11)	0.0220 (9)	0.0034 (9)
N1'	0.0574 (7)	0.0292 (6)	0.0654 (8)	0.0036 (6)	0.0122 (6)	−0.0013 (6)
N2'	0.0712 (10)	0.0674 (11)	0.0719 (9)	0.0242 (9)	0.0024 (8)	−0.0161 (8)
O1'	0.1246 (13)	0.0847 (11)	0.1221 (13)	0.0457 (10)	−0.0010 (10)	−0.0476 (11)
O2'	0.0779 (11)	0.1486 (18)	0.208 (2)	−0.0084 (12)	0.0644 (13)	−0.0781 (17)
O3'	0.0954 (9)	0.0313 (6)	0.0773 (8)	−0.0069 (6)	−0.0131 (7)	0.0029 (6)

### Geometric parameters (Å, º)

**Table:**

C1—C2	1.392 (3)	C1'—C2'	1.396 (3)
C1—C6	1.402 (2)	C1'—C6'	1.399 (2)
C1—C19	1.503 (3)	C1'—C19'	1.496 (2)
C2—C3	1.361 (3)	C2'—C3'	1.365 (3)
C2—H2	0.9300	C2'—H2'	0.9300
C3—C4	1.362 (3)	C3'—C4'	1.372 (3)
C3—H3	0.9300	C3'—H3'	0.9300
C4—C5	1.379 (2)	C4'—C5'	1.379 (3)
C4—H4	0.9300	C4'—H4'	0.9300
C5—C6	1.381 (2)	C5'—C6'	1.390 (2)
C5—H5	0.9300	C5'—H5'	0.9300
C6—C7	1.5183 (19)	C6'—C7'	1.521 (2)
C7—C8	1.542 (2)	C7'—C8'	1.535 (2)
C7—C10	1.5507 (19)	C7'—C10'	1.565 (2)
C7—H7	0.9800	C7'—H7'	0.9800
C8—N1	1.4666 (18)	C8'—N1'	1.456 (2)
C8—H8A	0.9700	C8'—H8'1	0.9700
C8—H8B	0.9700	C8'—H8'2	0.9700
C9—N1	1.4705 (19)	C9'—N1'	1.4724 (19)
C9—C13	1.5020 (19)	C9'—C13'	1.506 (2)
C9—C10	1.5234 (19)	C9'—C10'	1.531 (2)
C9—H9	0.9800	C9'—H9'	0.9800
C10—C11	1.510 (2)	C10'—C11'	1.507 (2)
C10—N2	1.516 (2)	C10'—N2'	1.522 (2)
C11—O3	1.4266 (17)	C11'—O3'	1.424 (2)
C11—H11A	0.9700	C11'—H11C	0.9700
C11—H11B	0.9700	C11'—H11D	0.9700
C12—O3	1.3679 (16)	C12'—O3'	1.3730 (19)
C12—C13	1.386 (2)	C12'—C17'	1.381 (2)
C12—C17	1.387 (2)	C12'—C13'	1.389 (2)
C13—C14	1.389 (2)	C13'—C14'	1.388 (2)
C14—C15	1.381 (2)	C14'—C15'	1.376 (3)
C14—H14	0.9300	C14'—H14'	0.9300
C15—C16	1.369 (3)	C15'—C16'	1.376 (3)
C15—H15	0.9300	C15'—H15'	0.9300
C16—C17	1.375 (2)	C16'—C17'	1.371 (3)
C16—H16	0.9300	C16'—H16'	0.9300
C17—H17	0.9300	C17'—H17'	0.9300
C18—N1	1.4576 (19)	C18'—N1'	1.454 (2)
C18—H18A	0.9600	C18'—H18D	0.9600
C18—H18B	0.9600	C18'—H18E	0.9600
C18—H18C	0.9600	C18'—H18F	0.9600
C19—H19A	0.9600	C19'—H19D	0.9600
C19—H19B	0.9600	C19'—H19E	0.9600
C19—H19C	0.9600	C19'—H19F	0.9600
N2—O1	1.207 (2)	N2'—O2'	1.176 (2)
N2—O2	1.226 (2)	N2'—O1'	1.198 (2)
			
C2—C1—C6	117.65 (19)	C2'—C1'—C6'	118.24 (16)
C2—C1—C19	119.08 (18)	C2'—C1'—C19'	118.69 (17)
C6—C1—C19	123.24 (16)	C6'—C1'—C19'	123.06 (17)
C3—C2—C1	122.31 (19)	C3'—C2'—C1'	122.21 (18)
C3—C2—H2	118.8	C3'—C2'—H2'	118.9
C1—C2—H2	118.8	C1'—C2'—H2'	118.9
C2—C3—C4	119.94 (18)	C2'—C3'—C4'	119.36 (19)
C2—C3—H3	120.0	C2'—C3'—H3'	120.3
C4—C3—H3	120.0	C4'—C3'—H3'	120.3
C3—C4—C5	119.4 (2)	C3'—C4'—C5'	120.05 (19)
C3—C4—H4	120.3	C3'—C4'—H4'	120.0
C5—C4—H4	120.3	C5'—C4'—H4'	120.0
C4—C5—C6	121.66 (18)	C4'—C5'—C6'	121.26 (17)
C4—C5—H5	119.2	C4'—C5'—H5'	119.4
C6—C5—H5	119.2	C6'—C5'—H5'	119.4
C5—C6—C1	119.01 (15)	C5'—C6'—C1'	118.87 (15)
C5—C6—C7	120.46 (13)	C5'—C6'—C7'	119.41 (14)
C1—C6—C7	120.51 (14)	C1'—C6'—C7'	121.70 (14)
C6—C7—C8	117.47 (13)	C6'—C7'—C8'	114.66 (13)
C6—C7—C10	115.17 (12)	C6'—C7'—C10'	115.53 (13)
C8—C7—C10	102.16 (11)	C8'—C7'—C10'	101.78 (12)
C6—C7—H7	107.1	C6'—C7'—H7'	108.2
C8—C7—H7	107.1	C8'—C7'—H7'	108.2
C10—C7—H7	107.1	C10'—C7'—H7'	108.2
N1—C8—C7	106.62 (12)	N1'—C8'—C7'	104.55 (13)
N1—C8—H8A	110.4	N1'—C8'—H8'1	110.8
C7—C8—H8A	110.4	C7'—C8'—H8'1	110.8
N1—C8—H8B	110.4	N1'—C8'—H8'2	110.8
C7—C8—H8B	110.4	C7'—C8'—H8'2	110.8
H8A—C8—H8B	108.6	H8'1—C8'—H8'2	108.9
N1—C9—C13	114.98 (11)	N1'—C9'—C13'	115.03 (12)
N1—C9—C10	99.98 (10)	N1'—C9'—C10'	99.63 (11)
C13—C9—C10	111.29 (12)	C13'—C9'—C10'	113.60 (12)
N1—C9—H9	110.1	N1'—C9'—H9'	109.4
C13—C9—H9	110.1	C13'—C9'—H9'	109.4
C10—C9—H9	110.1	C10'—C9'—H9'	109.4
C11—C10—N2	109.22 (13)	C11'—C10'—N2'	108.64 (14)
C11—C10—C9	109.73 (11)	C11'—C10'—C9'	108.88 (13)
N2—C10—C9	111.27 (12)	N2'—C10'—C9'	111.49 (12)
C11—C10—C7	115.32 (11)	C11'—C10'—C7'	114.13 (13)
N2—C10—C7	108.45 (11)	N2'—C10'—C7'	108.68 (13)
C9—C10—C7	102.74 (11)	C9'—C10'—C7'	105.03 (12)
O3—C11—C10	112.54 (11)	O3'—C11'—C10'	112.02 (13)
O3—C11—H11A	109.1	O3'—C11'—H11C	109.2
C10—C11—H11A	109.1	C10'—C11'—H11C	109.2
O3—C11—H11B	109.1	O3'—C11'—H11D	109.2
C10—C11—H11B	109.1	C10'—C11'—H11D	109.2
H11A—C11—H11B	107.8	H11C—C11'—H11D	107.9
O3—C12—C13	122.84 (12)	O3'—C12'—C17'	117.13 (14)
O3—C12—C17	115.78 (13)	O3'—C12'—C13'	121.77 (14)
C13—C12—C17	121.33 (13)	C17'—C12'—C13'	121.03 (15)
C12—C13—C14	117.51 (14)	C14'—C13'—C12'	117.46 (15)
C12—C13—C9	120.82 (12)	C14'—C13'—C9'	121.72 (14)
C14—C13—C9	121.55 (14)	C12'—C13'—C9'	120.74 (14)
C15—C14—C13	121.36 (17)	C15'—C14'—C13'	121.74 (17)
C15—C14—H14	119.3	C15'—C14'—H14'	119.1
C13—C14—H14	119.3	C13'—C14'—H14'	119.1
C16—C15—C14	119.96 (16)	C16'—C15'—C14'	119.55 (17)
C16—C15—H15	120.0	C16'—C15'—H15'	120.2
C14—C15—H15	120.0	C14'—C15'—H15'	120.2
C15—C16—C17	120.16 (16)	C17'—C16'—C15'	120.04 (18)
C15—C16—H16	119.9	C17'—C16'—H16'	120.0
C17—C16—H16	119.9	C15'—C16'—H16'	120.0
C16—C17—C12	119.64 (16)	C16'—C17'—C12'	120.13 (17)
C16—C17—H17	120.2	C16'—C17'—H17'	119.9
C12—C17—H17	120.2	C12'—C17'—H17'	119.9
N1—C18—H18A	109.5	N1'—C18'—H18D	109.5
N1—C18—H18B	109.5	N1'—C18'—H18E	109.5
H18A—C18—H18B	109.5	H18D—C18'—H18E	109.5
N1—C18—H18C	109.5	N1'—C18'—H18F	109.5
H18A—C18—H18C	109.5	H18D—C18'—H18F	109.5
H18B—C18—H18C	109.5	H18E—C18'—H18F	109.5
C1—C19—H19A	109.5	C1'—C19'—H19D	109.5
C1—C19—H19B	109.5	C1'—C19'—H19E	109.5
H19A—C19—H19B	109.5	H19D—C19'—H19E	109.5
C1—C19—H19C	109.5	C1'—C19'—H19F	109.5
H19A—C19—H19C	109.5	H19D—C19'—H19F	109.5
H19B—C19—H19C	109.5	H19E—C19'—H19F	109.5
C18—N1—C8	111.69 (12)	C18'—N1'—C8'	111.94 (13)
C18—N1—C9	114.03 (11)	C18'—N1'—C9'	114.55 (12)
C8—N1—C9	106.14 (11)	C8'—N1'—C9'	102.66 (12)
O1—N2—O2	123.89 (18)	O2'—N2'—O1'	122.81 (19)
O1—N2—C10	118.48 (17)	O2'—N2'—C10'	119.06 (17)
O2—N2—C10	117.53 (17)	O1'—N2'—C10'	118.12 (18)
C12—O3—C11	117.34 (11)	C12'—O3'—C11'	115.47 (12)
			
C6—C1—C2—C3	−1.2 (3)	C6'—C1'—C2'—C3'	1.2 (3)
C19—C1—C2—C3	176.7 (2)	C19'—C1'—C2'—C3'	−179.5 (2)
C1—C2—C3—C4	−1.0 (3)	C1'—C2'—C3'—C4'	−0.3 (4)
C2—C3—C4—C5	1.9 (3)	C2'—C3'—C4'—C5'	−0.6 (4)
C3—C4—C5—C6	−0.7 (3)	C3'—C4'—C5'—C6'	0.5 (4)
C4—C5—C6—C1	−1.5 (3)	C4'—C5'—C6'—C1'	0.5 (3)
C4—C5—C6—C7	176.76 (16)	C4'—C5'—C6'—C7'	−177.83 (19)
C2—C1—C6—C5	2.4 (2)	C2'—C1'—C6'—C5'	−1.3 (3)
C19—C1—C6—C5	−175.46 (17)	C19'—C1'—C6'—C5'	179.42 (17)
C2—C1—C6—C7	−175.85 (15)	C2'—C1'—C6'—C7'	177.01 (16)
C19—C1—C6—C7	6.3 (2)	C19'—C1'—C6'—C7'	−2.3 (2)
C5—C6—C7—C8	40.3 (2)	C5'—C6'—C7'—C8'	33.4 (2)
C1—C6—C7—C8	−141.51 (15)	C1'—C6'—C7'—C8'	−144.85 (15)
C5—C6—C7—C10	−80.15 (18)	C5'—C6'—C7'—C10'	−84.50 (19)
C1—C6—C7—C10	98.06 (16)	C1'—C6'—C7'—C10'	97.23 (18)
C6—C7—C8—N1	−131.16 (13)	C6'—C7'—C8'—N1'	−103.29 (15)
C10—C7—C8—N1	−4.12 (16)	C10'—C7'—C8'—N1'	22.18 (15)
N1—C9—C10—C11	77.41 (13)	N1'—C9'—C10'—C11'	86.74 (14)
C13—C9—C10—C11	−44.51 (16)	C13'—C9'—C10'—C11'	−36.11 (17)
N1—C9—C10—N2	−161.60 (11)	N1'—C9'—C10'—N2'	−153.42 (13)
C13—C9—C10—N2	76.48 (15)	C13'—C9'—C10'—N2'	83.73 (16)
N1—C9—C10—C7	−45.74 (13)	N1'—C9'—C10'—C7'	−35.89 (14)
C13—C9—C10—C7	−167.66 (11)	C13'—C9'—C10'—C7'	−158.74 (12)
C6—C7—C10—C11	39.70 (18)	C6'—C7'—C10'—C11'	14.51 (19)
C8—C7—C10—C11	−88.80 (15)	C8'—C7'—C10'—C11'	−110.37 (15)
C6—C7—C10—N2	−83.10 (16)	C6'—C7'—C10'—N2'	−106.91 (15)
C8—C7—C10—N2	148.40 (14)	C8'—C7'—C10'—N2'	128.21 (14)
C6—C7—C10—C9	159.04 (12)	C6'—C7'—C10'—C9'	133.68 (13)
C8—C7—C10—C9	30.53 (14)	C8'—C7'—C10'—C9'	8.79 (15)
N2—C10—C11—O3	−63.92 (15)	N2'—C10'—C11'—O3'	−62.29 (18)
C9—C10—C11—O3	58.29 (16)	C9'—C10'—C11'—O3'	59.30 (18)
C7—C10—C11—O3	173.69 (12)	C7'—C10'—C11'—O3'	176.27 (13)
O3—C12—C13—C14	174.98 (15)	O3'—C12'—C13'—C14'	176.47 (15)
C17—C12—C13—C14	−2.1 (2)	C17'—C12'—C13'—C14'	−0.5 (2)
O3—C12—C13—C9	−0.9 (2)	O3'—C12'—C13'—C9'	−0.1 (2)
C17—C12—C13—C9	−177.98 (14)	C17'—C12'—C13'—C9'	−177.13 (15)
N1—C9—C13—C12	−94.95 (16)	N1'—C9'—C13'—C14'	78.02 (18)
C10—C9—C13—C12	17.80 (19)	C10'—C9'—C13'—C14'	−168.05 (14)
N1—C9—C13—C14	89.33 (18)	N1'—C9'—C13'—C12'	−105.52 (16)
C10—C9—C13—C14	−157.91 (15)	C10'—C9'—C13'—C12'	8.41 (19)
C12—C13—C14—C15	2.6 (3)	C12'—C13'—C14'—C15'	2.1 (2)
C9—C13—C14—C15	178.41 (17)	C9'—C13'—C14'—C15'	178.67 (15)
C13—C14—C15—C16	−1.3 (3)	C13'—C14'—C15'—C16'	−2.1 (3)
C14—C15—C16—C17	−0.5 (3)	C14'—C15'—C16'—C17'	0.4 (3)
C15—C16—C17—C12	1.0 (3)	C15'—C16'—C17'—C12'	1.1 (3)
O3—C12—C17—C16	−176.89 (16)	O3'—C12'—C17'—C16'	−178.20 (16)
C13—C12—C17—C16	0.4 (3)	C13'—C12'—C17'—C16'	−1.1 (3)
C7—C8—N1—C18	−150.02 (13)	C7'—C8'—N1'—C18'	−170.11 (13)
C7—C8—N1—C9	−25.18 (15)	C7'—C8'—N1'—C9'	−46.77 (15)
C13—C9—N1—C18	−73.23 (15)	C13'—C9'—N1'—C18'	−65.66 (17)
C10—C9—N1—C18	167.51 (12)	C10'—C9'—N1'—C18'	172.51 (13)
C13—C9—N1—C8	163.38 (12)	C13'—C9'—N1'—C8'	172.76 (12)
C10—C9—N1—C8	44.12 (13)	C10'—C9'—N1'—C8'	50.93 (14)
C11—C10—N2—O1	−27.39 (18)	C11'—C10'—N2'—O2'	150.5 (2)
C9—C10—N2—O1	−148.68 (15)	C9'—C10'—N2'—O2'	30.5 (3)
C7—C10—N2—O1	99.03 (17)	C7'—C10'—N2'—O2'	−84.8 (2)
C11—C10—N2—O2	156.01 (14)	C11'—C10'—N2'—O1'	−30.5 (2)
C9—C10—N2—O2	34.72 (18)	C9'—C10'—N2'—O1'	−150.48 (17)
C7—C10—N2—O2	−77.57 (16)	C7'—C10'—N2'—O1'	94.22 (19)
C13—C12—O3—C11	13.4 (2)	C17'—C12'—O3'—C11'	−160.04 (15)
C17—C12—O3—C11	−169.35 (14)	C13'—C12'—O3'—C11'	22.9 (2)
C10—C11—O3—C12	−42.60 (18)	C10'—C11'—O3'—C12'	−53.5 (2)

### Hydrogen-bond geometry (Å, º)

*Cg*11 and *Cg*3 are the centroids of the C12'–C17' and C1–C6 rings, respectively.

**Table:**

*D*—H···*A*	*D*—H	H···*A*	*D*···*A*	*D*—H···*A*
C15—H15···O1′^i^	0.93	2.60	3.282 (3)	131
C4′—H4′···O2^ii^	0.93	2.56	3.407 (3)	152
C14′—H14′···O2	0.93	2.59	3.327 (2)	136
C19′—H19*F*···O1′	0.96	2.54	3.391 (3)	148
C18—H18*A*···*Cg*11^i^	0.96	2.92	3.854 (2)	166
C19′—H19*E*···*Cg*3^iii^	0.96	2.94	3.811 (3)	152

Symmetry codes: (i) *x*, *y*−1, *z*; (ii) −*x*+1, −*y*+1, −*z*; (iii) *x*−1/2, −*y*−1/2, *z*−1/2.
